# Exploiting defective RRAM array as synapses of HTM spatial pooler with boost-factor adjustment scheme for defect-tolerant neuromorphic systems

**DOI:** 10.1038/s41598-020-68547-5

**Published:** 2020-07-16

**Authors:** Jiyong Woo, Tien Van Nguyen, Jeong Hun Kim, Jong-Pil Im, Solyee Im, Yeriaron Kim, Kyeong-Sik Min, Seung Eon Moon

**Affiliations:** 1 ICT Creative Research Laboratory, Electronics and Telecommunications Research Institute, Daejeon, 34129 South Korea; 2 School of Electrical Engineering, Kookmin University, Seoul, 02707 South Korea

**Keywords:** Electrical and electronic engineering, Electronic devices

## Abstract

A crossbar array architecture employing resistive switching memory (RRAM) as a synaptic element accelerates vector–matrix multiplication in a parallel fashion, enabling energy-efficient pattern recognition. To implement the function of the synapse in the RRAM, multilevel resistance states are required. More importantly, a large on/off ratio of the RRAM should be preferentially obtained to ensure a reasonable margin between each state taking into account the inevitable variability caused by the inherent switching mechanism. The on/off ratio is basically adjusted in two ways by modulating measurement conditions such as compliance current or voltage pulses modulation. The latter technique is not only more suitable for practical systems, but also can achieve multiple states in low current range. However, at the expense of applying a high negative voltage aimed at enlarging the on/off ratio, a breakdown of the RRAM occurs unexpectedly. This stuck-at-short fault of the RRAM adversely affects the recognition process based on reading and judging each column current changed by the multiplication of the input voltage and resistance of the RRAM in the array, degrading the accuracy. To address this challenge, we introduce a boost-factor adjustment technique as a fault-tolerant scheme based on simple circuitry that eliminates the additional process to identify specific locations of the failed RRAMs in the array. Spectre circuit simulation is performed to verify the effect of the scheme on Modified National Institute of Standards and Technology dataset using convolutional neural networks in non-ideal crossbar arrays, where experimentally observed imperfective RRAMs are configured. Our results show that the recognition accuracy can be maintained similar to the ideal case because the interruption of the failure is suppressed by the scheme.

## Introduction

Neuromorphic computing technology that emulates the role and function of the human brain into electronic systems has received great attention recently^1^. This is because data processing is dramatically speeded up by the brain structure, where numerous neurons are connected by synapses in parallel. The bio-inspired parallel operation has been thus exploited widely in various fields by developing neural network algorithms in software^2^. The algorithms are specifically useful for new industrial applications such as autonomous vehicles and drones that need to process massive amounts of data in real time. As vector–matrix multiplication (VMM) plays an important role in realizing the parallel operation in the algorithms, this simple but time-consuming computation step should be demonstrated in hardware^3^. The VMM can be implemented in conventional computing systems such as central and graphics processing units^4, 5^, but it becomes energy inefficient against expectations. Since the system is based on Von-Neumann architecture, the results computed at processing units must be transferred to memory to store. The frequently moving data between the processing unit and memory takes a lot of time and power, which is known as the Von-Neumann bottleneck^6^.

In this regard, a crossbar array architecture, where word lines and bit lines are located perpendicularly, has been considered to speed up the VMM^7^. In this configuration, data corresponding to synaptic weight is stored at each intersection. When signals in the form of voltage enter the word lines simultaneously, the multiplication of the voltage and synaptic weight at each cross in the array is performed. The calculated results are then summed along the bit line, and a single column current is shown at the end of the bit line. Note that the VMM takes place at the location, where the weights are physically stored, thereby saving time by avoiding the Von-Neumann bottleneck. In order to maximize the functionality and versatility of the VMM in the crossbar array based neuromorphic systems, an appropriate synaptic weight element should be utilized^8^. In particular, a capability to have multilevel weights in highly scalable devices is strongly required. To date, various emerging devices such as phase change memory^9^, spin-transfer torque magnetic memory^10^, ferroelectric memory^11^, electrochemical memory^12^, and resistive switching memory (RRAM)^13–19^ have been suggested for the synaptic devices. Among them, the RRAM driven by formation and rupture of a filament has been considered as the most promising candidate due to its excellent scalability (< 10 nm) and multilevel operation^17^. The RRAM devices have been implemented with the crossbar array architecture in the form of one-transistor (or one-selector) and one-RRAM configuration. This structure can thus minimize disturbances due to unwanted sneak-path currents from neighboring cells while writing and reading only the selected RRAM^20,21^.

**Figure 1:**
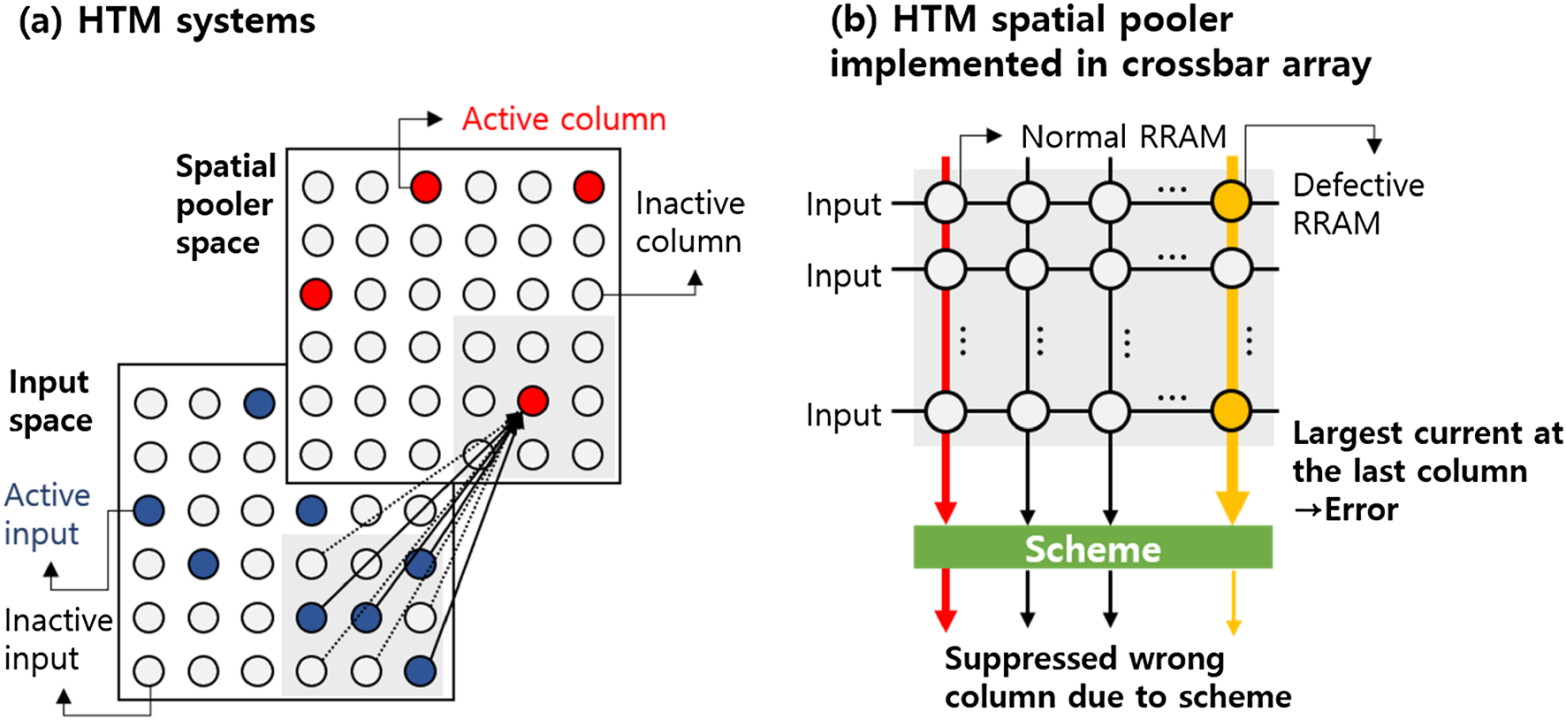
(**a**) HTM system consists of an input space and a spatial pooler space. (**b**) The HTM spatial pooler can be implemented in the crossbar array. In real crossbar arrays, defective RRAMs are included. As a result, the largest current is calculated in the unpredictable column. Additional schemes are required to intentionally disable the wrong column.

While most studies have focused on achieving the multilevel states^13–19^, reliability issues of the multiple states that play a crucial role in the VMM from operational perspective have been rarely explored^22,23^. More importantly, a device failure in the crossbar RRAM array causes significant performance degradation in the neuromorphic systems^24,25^. Among the various neuromorphic architectures, the crossbar array can be served as a spatial pooler (SP) for hierarchical temporal memory (HTM) that describes the functionality of the human neocortex^26^, as shown in Fig. 1. One of the important roles in the SP structure is to encode information by converting input data through the synapses to a sparse distributed representation. For the given input, the assigned weights of the analog synapses are added. The output is then activated only when the weighted sum exceeds a threshold, and the sparse binary pattern is generated. Recently, beyond the mixed analog–digital SP structure, the design and implementation of a fully analog SP has been attempted^27^.

Unlike memory operations that select specific word line and bit line to read only one RRAM, all the column currents are read simultaneously during the pattern recognition process. When stuck-at-short faults occur in multiple RRAMs in a particular column, the column current is primarily governed by the failed RRAMs, no matter how the input vectors and weights are configured. As shown in Fig. 1b, instead of the first column designed to represent the largest column, the wrong column is often activated, resulting in a recognition error. This problem was overcome by using a conventional fault-aware mapping scheme that diagnoses the location of the failed devices^28^. The VMM was then performed after the rearranged weight pattern was mapped to the array. However, the drawback is that complicated circuits and capacitors required to read and verify all RRAMs occupy a large area. To minimize the effect of the failed RRAMs without using the fault-aware mapping, we introduced a boost-adjustment technique implemented with simple circuits proposed in the previous work^25^. However, the types of the failures that can be specifically observed in real devices dedicated to the synaptic element have not been discussed.

Therefore, in this study, we first showed the stuck-at-short fault in the RRAM, one of primary failures, at the expense of the large on/off ratio. The scheme was then validated by using the resistance values, its distributions, and failure rates obtained from the experimental results of the RRAMs as simulation parameters. The pattern recognition accuracy of the Modified National Institute of Standards and Technology (MNIST) dataset was evaluated.

## Results and discussion

**Figure 2:**
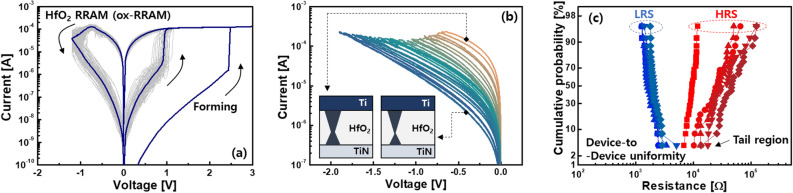
(**a**) The I–V characteristics of the ox-RRAM. (**b**) By increasing the negative voltage during the reset process, the current was analogously decreased. (**c**) Resistance distributions of the LRS and HRS of the ox-RRAM.

**Figure 3:**
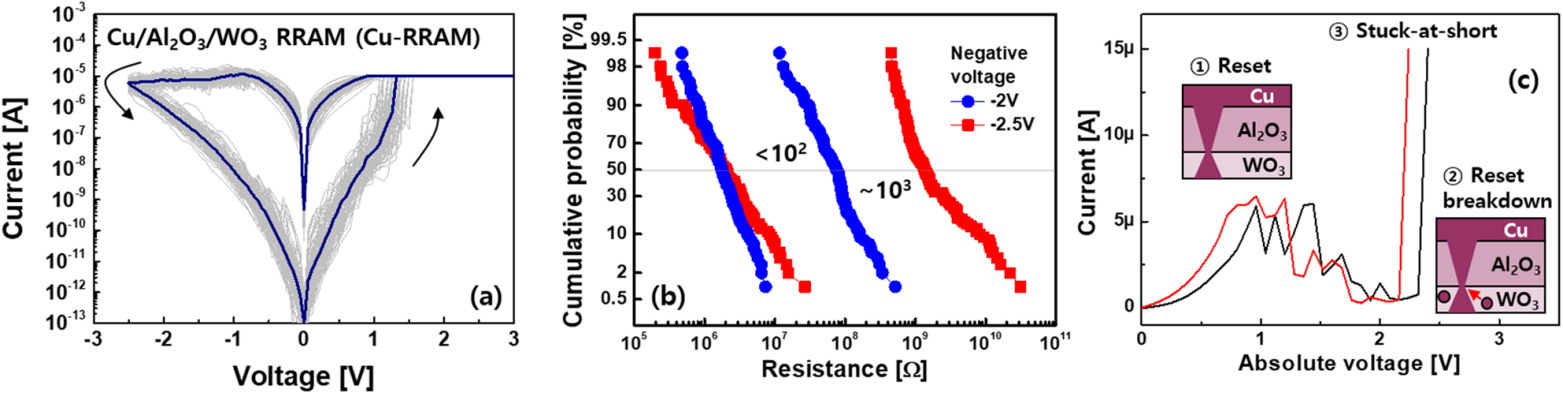
(**a**) The I–V characteristics of the Cu-RRAM. The larger on/off ratio of the Cu-RRAM was noticeable compared to the ox-RRAM (Fig. 2a). (**b**) Similar to Fig. 2b, the increased negative voltage was used to obtain the larger on/off ratio. (**c**) The breakdown occurred unexpectedly during the reset process.

**Figure 4:**
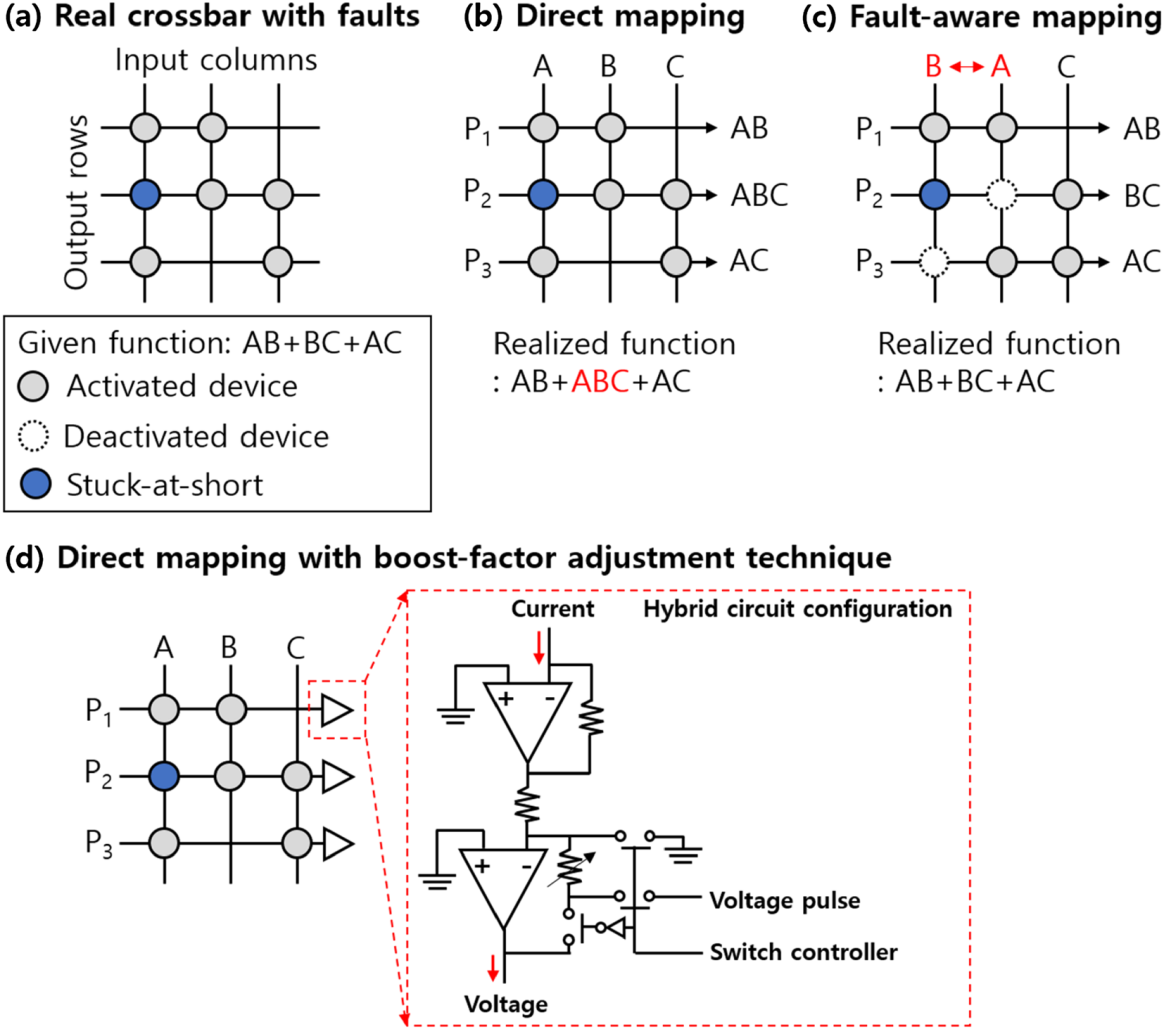
(**a**) Non-ideal crossbar with faults. (**b**) Direct mapping to realize the given function. (**c**) Fault-aware mapping considering the failure for performing AB + BC + AC. (**d**) Direct mapping using the boost-factor adjustment manner to suppress the impact of the failure. The simplified schematic diagram of the hybrid circuit was shown in the box, and detailed operation was described in the reference^25^. Here a column with a large number of defective RRAMs was less activated by the boost-factor adjustment^25^.

Typical DC current–voltage (I–V) characteristics of a HfO_2_ based RRAM with TiN/Ti/HfO_2_/TiN stack^14^ defined as an ox-RRAM were shown in Fig. 2a. When a positive voltage was applied to the ox-RRAM, oxygen vacancies generated in the HfO_2_ were bridged between top and bottom electrodes, creating a conductive filament. As a result, an abruptly increased current was shown through the formed filament, which is a low resistance state (LRS). On the other hand, a negative voltage caused the vacancies to move away from the filament during the reset process, resulting in a high resistance state (HRS). Here, note that the gradual I–V transition was shown in the negative voltage region. It has been described that the weakest constriction part of the filament is reduced based on the hourglass model^29^ rather than the complete disconnection of the filament usually showing an abrupt transition. Because the filament was incompletely disconnected, the oxygen vacancies were needed to travel only small distances for the next set operation. The subsequent set voltage was lower than the initial forming voltage in proportion to the oxide thickness^30^. Based on this working principle, multiple weight states in the form of resistance values were achieved by controlling either current limiter^18^ and energy supplied to the RRAM^31,32^ during the set and reset, respectively. Adjusting the current limiter determined the maximum allowable current relevant to the filament size. Elaborately releasing the limiter resulted in the multilevel states in the low resistance range^18^, but the power consumption increased. Whereas, sweeping with progressively increasing negative voltage steadily decreased the width. In order for oxygen vacancies to overcome activation energy, sufficient time as well as the voltage must be provided to the RRAM. That is, the analogously modulated multiple states in the high resistance range, which can be a more energy-efficient approach, were achieved by the energy controlled by the amplitude and width of voltage, as shown in Fig. 2b. Here, it is preferable to obtain an RRAM with a large on/off ratio, taking into account the case, where each state can be overlapped by the stochastic nature of the ion migration^33,34^. Assuming that the number of achievable states is the same in the RRAM, the large on/off ratio ensures a reasonable margin between the states despite the inherent variability. Considering the binary states for simplicity, the LRS resistance (R_LRS_) and HRS resistance (R_HRS_) seemed to be clearly distinguished in the single ox-RRAM (Fig. 2a). The stability for each state at high temperature^35^ and repeated cycles^36^ was confirmed. However, the difference in resistance was affected by the device-to-device variation, and each state might easily be overlapped in the tail region, as shown in Fig. 2c. The insufficient margin made peripheral circuits (e.g. analog-to-digital converter and sense amplifier) connected to the crossbar array difficult to sense the states accurately, thereby degrading the recognition accuracy^8^. Although innovative device engineering^37,38^ has been suggested to improve the uniformity, this problem seemed to be inevitable due to the random ionic motion in materials. Since it is difficult to completely exclude the variability, an alternative approach was to increase the on/off ratio of the RRAM so that the non-uniform resistance distributions can be ignored^39^. That is, Cu or Ag instead of the oxygen vacancies was used for the filament source^40^. An RRAM device with Cu/TiW/Al_2_O_3_/WO_3_/W stack^41^ denoted as a Cu-RRAM was studied as an example in this study. During the set operation, a rapid transition of the I–V curve was shown, while the gradual reset operation was observed in the reset. The three-dimensional observation of the filament revealed by in-situ conducting atomic force microscopic study^42^ means that the switching characteristics can be described identically by using the aforementioned physical mechanism^6,8,13^. Compared to the ox-RRAM, the filament in the Cu-RRAM consisted mostly of Cu ions. This made the device immune to the external stress, resulting in stable noise and disturbance properties^43^. As shown in Fig. 3a, it was noticeable that a much larger on/off ratio was achieved in the Cu-based RRAM than the ox-RRAM. It has been explained that the sources of the filament in the Cu-based RRAM are provided from the external Cu electrode as an ion reservoir rather than the oxide medium by breaking the bond with the oxygen during the forming process^39^. Thus, the less destructed oxide allowed the higher R_HRS_, resulting in the on/off ratio greater than > 10^3^. When the voltage pulse of − 2 V with a width of 100 ns was applied to the LRS of the Cu-RRAM, the HRS read at − 0.1 V was shown in the range of tens to hundreds of MΩ. The large negative voltage pulse of − 2.5 V further increased the R_HRS_, enlarging the on/off ratio of ~ 10^3^ in pulse operation. These results are expected to tolerate the inevitable variability of the Cu-RRAM. In addition, the large on/off ratio is beneficial for achieving higher bit-precision, which can increase recognition accuracy^44^. However, the application of the large negative voltage caused a permanent breakdown of the Cu-RRAM during the reset process, as shown in the I–V curves (Fig. 3c). As the negative voltage was increased, the current began to gradually decrease, then increased unexpectedly. We measured 15 Cu-RRAMs, and 7 of the 15 devices showed the breakdown in the DC I–V characterization, as shown in Fig. 3c. 10% probability of the failure was also observed in the AC pulse cycling (not shown here)^41^. These results meant that there was a trade-off relationship between the large on/off ratio and failure. Since this failure was observed even in the ox-RRAM^45^, it has been generally understood that the constricted part was strengthened due to the residual Cu ions or oxygen vacancies. Here, it is worthy to note that the permanent failure must be considered importantly in the neuromorphic systems that sense the column current from the crossbar array and generate a signal to activate the next devices for pattern recognition. Ideally, specific resistance values are assigned to each Cu-RRAM in the array so that a large current is calculated at a predetermined column when certain input voltages come in. When the column consists of the Cu-RRAMs with the stuck-at-short, much larger current than expected is observed. As a result, it becomes difficult to classify the column current that should be solely changed by the input voltages and weight patterns, thereby worsening the recognition accuracy. Considering that a Boolean function of AB + BC + AC needs to be executed for the recognition (Fig. 4a), the weight map is implemented directly on the crossbar array, as shown in Fig. 4b. The stuck-at-short located in the second row interrupts to address the correct product (P), and the wrong function of AB + ABC + AC is eventually realized through an AND gate located at the end of the row^46^. The problem related to the stuck-at-short in the neuromorphic systems can be compensated by the fault-aware defect mapping scheme, as shown in Fig. 4c. In this scheme, before transferring the resistances to the array, a diagnosis to detect the failure is performed to identify the defect map of the failed devices. Then, a new map that relocates the activated devices to minimize the error is applied. The information in the row can be also switched between A and B to demonstrate the given function correctly. However, the fault-aware defect mapping scheme needs to use the complex digital circuits occupying a large layout area^28^. We thus developed the boost-factor adjustment scheme, where the erroneous neurons connected with many defective synapses can be suppressed in a self-controlled manner without using the defect map measured during the previous diagnostic process^25^. The boost-factor adjustment scheme suppressed the gain of the current-to-voltage converter, when the column was activated frequently due to many failures. Figure 4d showed a schematic diagram of the boost-adjustment scheme consisting of diode-connected metal-oxide semiconductor field effect transistors (MOSFETs), comparators, and variable resistor. The SP algorithm is defined in four phases. In initialization (phase 1), a certain number of columns are selected first to receive the input data. To identify how many synapses are connected to each column at the given input, the boosting factor is multiplied in overlap step (phase 2). The factor, which is a dynamic value, indicates how often the column is activated compared to adjacent columns. Through inhibition stage (phase 3), the winning column becomes active, and the other columns are inhibited. Then, Hebbian learning rule is performed to update the synaptic weights up or down (phase 4). In phase 3, when the column current exceeds a threshold at a given input vector, the column is activated. The degree of the activation (α) for the column i is defined as follow:

1 $${\alpha } = \frac{1}{M}\mathop \sum \limits_{j = 1}^{M} \left( {activation\;\left( {column\;i} \right)} \right).$$

M is the number of test vectors. According to Eq. (1), the activation function of column i becomes 1, if the column is activated, otherwise it is 0. If the column is activated frequently, the activation may be incorrect due to the large number of failed RRAMs. In this case, the boost-factor (β) can be adjusted to suppress the abnormally frequent activation, which can be simply defined as follows:

2 $$\beta = e^{{ - B\left( {\alpha_{i} } \right)}}$$

where the B is a positive parameter that controls the strength of the adaptation effect. Thus, lowering the β, which was inversely related to α, can reduce the false activation, minimizing the loss of recognition accuracy. The summed current of each column was shown in the form of the voltage through the current-to-voltage converter at the end of the column. The converted voltage reached the hybrid circuit configuration of variable resistor and diode-connected MOSFET area for boost-factor adjustment. According to the activity ratio of the column, the resistance of the variable resistor was changed by applied pulses through the boosting factor adjustment controller. The voltage multiplied by the adjusted boosting factor was then compared to reference voltage of the comparator. When the voltage of the column was close to the reference, the column started to be activated as a winner, while simultaneously inhibiting neighboring columns. To select the winning column, the reference voltage was obtained from the diode-connected MOSFETs by extracting the largest output voltage among adjacent columns. Therefore, the hybrid circuit was able to reduce the occupied area because it does not use a capacitor that represents the voltage based on the accumulated amount of charge.

**Figure 5:**
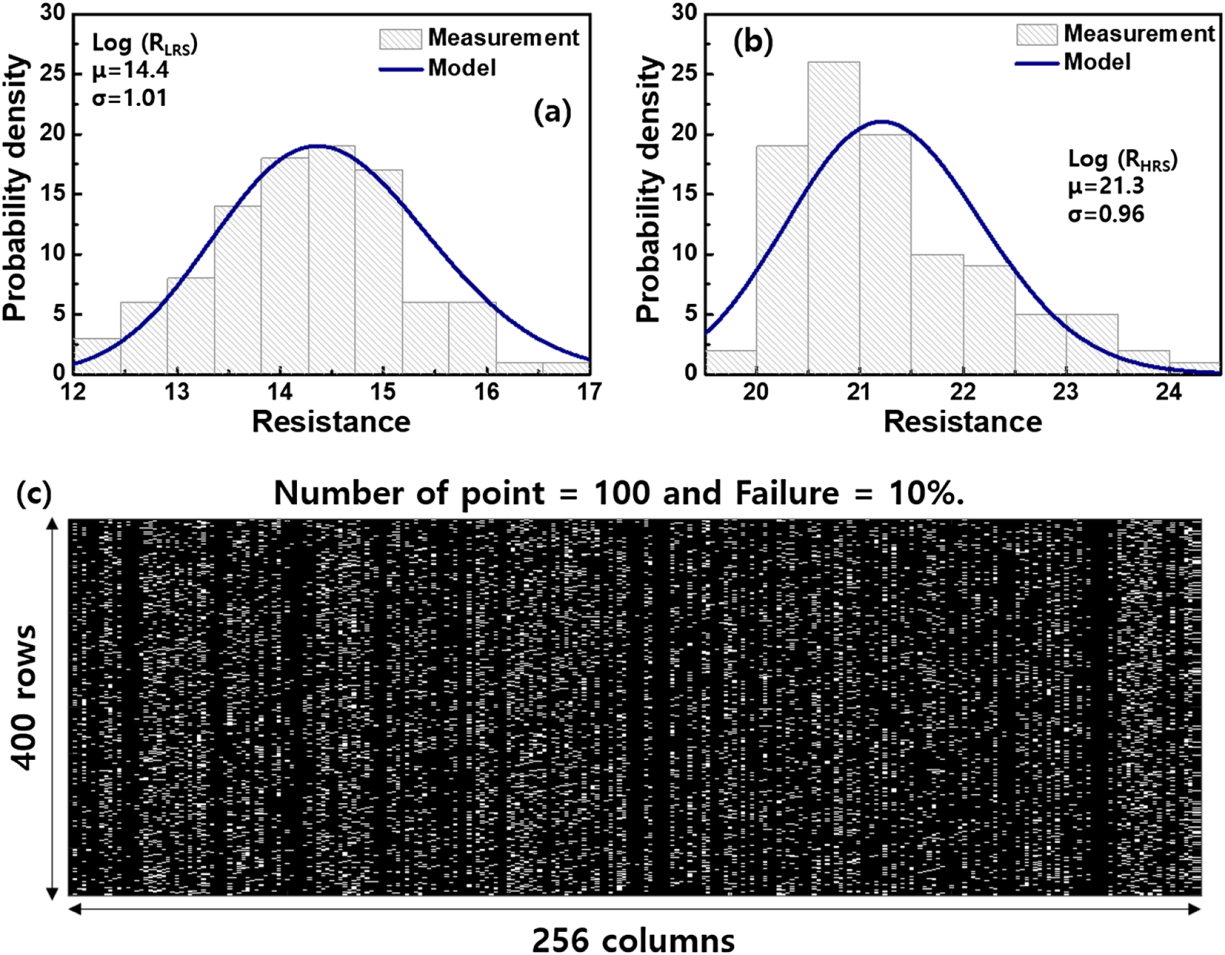
(**a**) The relationship of log (R_LRS_) and probability density (%) with log-normal distribution. Here the number of samples measured is 100 (%). (**b**) The relationship of log (R_HRS_) and probability density (%) with log-normal distribution. Here the number of samples measured is 100. (**c**) The failure map of the non-ideal 400 × 256 crossbar array consisting of the obtained R_LRS_ and R_HRS_. The defective devices were randomly distributed and the failure percentage of the RRAM was assumed 10%.

On the basis of the experimentally obtained resistance values, the designed scheme was validated by evaluating the accuracy of the MNIST handwritten dataset in simulation. As shown in Fig. 5a, the log-normal distributions of the R_LRS_ and R_HRS_ of the Cu-RRAMs were simulated by the following equation, and the average values (*μ*) of 14.4 and 21.3 were first extracted.

3 $$y = f\left( {\mu ,\sigma } \right) = \frac{1}{{x\sigma \sqrt {2\pi } }}exp\left\{ {\frac{{ - \left( {loglog x - \mu } \right)^{2} }}{{2\sigma^{2} }}} \right\},\quad{\text{ for}}\,\,\,x > \, 0.$$

For the MNIST vector consisting of 20 × 20 pixels in convolutional neural networks, the number of input voltages was 400. The I–V relationship of the RRAM was simulated by a behavioral model using Verilog-A in CADENCE Spectre for circuit simulation. The MNIST recognition was simulated in MATLAB software. The 400 × 256 crossbar array with randomly distributed μ of the R_LRS_ and R_HRS_ was constructed. Parasitic components such as the source and neuron resistances assumed to be 0.27% and 0.067% of HRS were also considered. The generated netlist was solved by MATLAB to calculate the column current in the array. It was assumed that experimentally observed 10% change of the faults occurred in the array. The probability was considered reasonable because the 10% was also used in the literature reporting the mapping strategy^47^. The standard deviations (*σ*) of 1 obtained from the cycle-to-cycle variation test was adopted. To understand how the column’s activation affects the recognition accuracy, the column number was increased to 1,024 and 4,096 at the given number of rows.

Table 1 shows the MNIST recognition accuracy in the non-ideal crossbar array. At the given columns of 256, the Cu-RRAM array with variations without failure exhibited accuracy of about 78%, which is the closest to the ideal case. It means that the neural network consisting of the Cu-RRAM with the large on/off ratio was somewhat resilient to the fluctuations. The large column current due to the failure that occurred was undesirably multiplied by the large boosting factor, so the recognition accuracy was reduced by half. The degraded accuracy can be improved by defect-aware algorithms, but the time to run the algorithms and the area became a major drawback. However, even if the failure was additionally introduced, the recognition accuracy was maintained above 75% by the scheme. This tendency was similarly observed when the number of columns was increased. The accuracy was further improved by increasing the column. This is because more detailed features can be extracted from the MNIST dataset and stored in the increased columns. The enhanced resolution in the output resulted in the improved accuracy. In addition, assuming that the energy to program the crossbar RRAM array was about 4 mJ at a given the number of the RRAMs in the column, the additional energy of 1,000 times less to run the boost-factor adjustment technique was needed^48,49^. This was because the RRAM and other components, which were key components in the hybrid circuit, can be operated quickly, thereby reducing running time as well as area demanded to implement the algorithms.

**Table 1:** Comparison of the recognition accuracy on the MNIST dataset as a function of the number of columns, standard deviation of log (HRS) and log (LRS), and stuck-at-fault (%).

# of columns	Standard deviation (σ)	Probability of stuck-at-short (%)	Recognition accuracy (%)	
			w/o boost factor adjustment	w/ boost factor adjustment
256	1	0	77.9	77.9
	0	10	40.6	76.57
	1	10	37.4	76.57
1,024	1	0	92.6	92.6
	0	10	55.3	91.3
	1	10	51.2	91.1
4,096	1	0	95.4	95.4
	0	10	64.3	95.2
	1	10	61.2	94.5

## Conclusion

In this study, we exploited the Cu-RRAM with the large on/off ratio as the synaptic element for the crossbar array based neuromorphic systems. Even if inherent variability originated from the stochastic nature of the ion motion was shown, the large R_LRS_ and R_HRS_ difference of the Cu-RRAM was expected to help to achieve sufficient margin. However, as the trade-off relationship, the stuck-at-short fault was inevitably observed during the device operation. The column current in the Cu-RRAM array was thus determined by the failure, not the desired product of input voltage and resistance of the Cu-RRAM, degrading the recognition accuracy. To minimize the effect of the failure, we utilized the fault-tolerant scheme implemented by the hybrid circuit to lower the erroneously activated columns by the newly adjusted boost-factor. The scheme was validated in circuit simulation by evaluating the recognition accuracy on the MNIST dataset. The simulation results showed that the recognition accuracy of the boost-factor adjusted crossbar array with the *σ* of 1 and failure of 10% was similar to the ideal case. Therefore, we believe that the use of Cu-RRAM and boost-factor adjustment as appropriate synaptic element and optimized scheme respectively can demonstrate the multilevel states and failure compensation simultaneously for highly accurate neuromorphic systems.
